# Genotype and Environment Affect the Grain Quality and Yield of Winter Oats (*Avena sativa* L.)

**DOI:** 10.3390/foods10102356

**Published:** 2021-10-03

**Authors:** Catherine J. Howarth, Pilar M. J. Martinez-Martin, Alexander A. Cowan, Irene M. Griffiths, Ruth Sanderson, Susan J. Lister, Tim Langdon, Sarah Clarke, Nick Fradgley, Athole H. Marshall

**Affiliations:** 1 Institute of Biological, Environmental and Rural Sciences, Aberystwyth University, Gogerddan, Aberystwyth SY23 3EE, Ceredigion, UK; pilar.mmartin.83@gmail.com (P.M.J.M.-M.); syc@aber.ac.uk (A.A.C.); igg@aber.ac.uk (I.M.G.); rts@aber.ac.uk (R.S.); sll@aber.ac.uk (S.J.L.); ttl@aber.ac.uk (T.L.); thm@aber.ac.uk (A.H.M.); 2 ADAS Gleadthorpe, Mansfield NG20 9PD, Nottinghamshire, UK; Sarah.Clarke@adas.co.uk; 3 Organic Research Centre, Elm Farm, Hamstead Marshall, Newbury RG20 0HR, Berkshire, UK; Nick.Fradgley@niab.com

**Keywords:** oats, quality, β-glucan, grain size, yield, G × E interaction, image analysis, milling

## Abstract

The extent to which the quality and yield of plant varieties are influenced by the environment is important for their successful uptake by end users particularly as climatic fluctuations are resulting in environments that are highly variable from one growing season to another. The genotype-by-environment interaction (GEI) of milling quality and yield was studied using four winter oat varieties in multi-locational trials over 4 years in the U.K. Significant differences across the 22 environments were found between physical grain quality and composition as well as grain yield, with the environment having a significant effect on all of the traits measured. Grain yield was closely related to grain number m^−2^ whereas milling quality traits were related to grain size attributes. Considerable genotype by environment interaction was obtained for all grain quality traits and stability analysis revealed that the variety Mascani was the least sensitive to the environment for all milling quality traits measured whereas the variety Balado was the most sensitive. Examination of environmental conditions at specific within-year stages of crop development indicated that both temperature and rainfall during grain development were correlated with grain yield and β-glucan content and with the ease of removing the hull (hullability).

## 1. Introduction

Oats (*Avena sativa* L.) are a high quality cereal, currently experiencing resurgence in its use for human consumption [1], due to the recognized health benefits attributed to the nutritional qualities of the oat grain [2,3]. Dietary benefits associated with phytochemicals within the oat grain, such as β-glucan, and approved health claims for oat β-glucan has contributed to the increased interest in oats as a food ingredient and led to incorporation into an increasing number of food products [4]. As the interest in oat products increases, so the demand for raw materials with particular health benefits requires oat breeding programs aimed at the release of improved oat cultivars which meet the needs of food industries [5] and at the same time have the milling qualities which are required to ensure their uptake by the milling industry.

Grain quality and yield determine much of the value of an oat crop to the producer with several grain characteristics routinely used to define milling quality [6]. Although alternative methods for quantifying grain milling quality through detailed analysis of grain size and shape using image analysis are now being developed [1], oats for milling are currently traded on the basis of their hectoliter weight, screenings and a subjective assessment of condition [7]. Hectoliter weight (often referred to as specific weight or test weight) is a measure of grain density and although regarded as a poor indicator of milling quality [8] is still routinely used in analyzing oat crops ex farm. Groat content and ease of hull removal (hullability) are the most important traits for milling quality as groat content is the characteristic most closely associated with the millers extract yield of product [7,8] and hullability has important implications for mill efficiency. Varieties with poor hullability require greater impact speeds within the dehuller during the milling process and result in greater groat breakage, thereby depressing the miller’s extract yield. Poor hullability also reduces mill efficiency, increasing cost and energy usage as well as delaying product flow. Improving the physical characteristics of the oat grain to maximize milling yield has become a major target of many oat breeding programs [1] and therefore understanding the genetic and environmental effect on these characteristics is increasingly important.

Developing new oat varieties that combine high yield, enhanced β-glucan content and high groat content has proven challenging such that improvement in one trait tends to be accompanied by a reduction in the others [9,10]. Plant breeders also aim to develop improved crop varieties that are adapted to produce high yields of quality grain over a wide range of environments [6] with the adaptability of a variety usually tested by the degree of interaction with different environments under which it is planted [11]. Analysis of the genotype x environment interaction (GEI) on grain yield and quality is therefore essential in variety evaluation [12,13] and to understand the adaptability and stability of varieties [14] for different environments. The GEI effects on selected oat grain quality traits [6,11], and on β-glucan content in commercially available varieties [2,15] and within related wild species [5] have been studied, but there is limited information on other grain components. There is also limited published information on the GEI on grain physical quality traits of importance to the milling industry or for those compositional traits important for human and animal consumption.

The present paper reports a study of the grain quality, grain composition grain yield of four commercially available winter oat varieties grown in 9 locations over 4 harvest years, enabling the GEI to be quantified and the stability of the four oat varieties over different environments to be analyzed. The effect of environment on grain quality traits related to milling efficiency and feed value is crucial for the milling, food and feed sector to predict the performance of different varieties in different environments.

## 2. Materials and Methods

### 2.1. Plant Material, Field Sites and Plot Management

The study included four commercially available winter oat varieties from the Aberystwyth University winter oat breeding program including two of the most widely grown winter oat varieties in the UK over the last 20 years, Gerald and Mascani, grown in replicated field trials at 9 sites across the UK between 2010 and 2014 (Table 1). Sites were chosen to represent contrasting environmental conditions within the UK and included the geographical areas where oats are grown in arable rotations. The 22 site-harvest year combinations are subsequently referred to as environments. Each trial included at least three replicate plots (1.8 × 6 m) of each variety, sown in a randomized block design, at a sowing rate of 300 seeds m^−2^ except for at ORC Elm farm where a sowing rate of 425 seeds m^−2^ was used as commonly used in organic practices. The trials were sown between September and October at all sites except for one environment where conditions delayed sowing until the following spring (Table 1). Fungicides, weed control and fertilizer followed the established protocols used for Recommend List testing of varieties in the UK [16] except at ORC Elm Farm which was managed organically as previously described [17].

### 2.2. Climatic Conditions at Each Site

Daily rainfall (mm), minimum and maximum temperatures (°C) and relative humidity (%) were recorded for each site using in-field weather stations where available or using publically available data from local meteorological office stations [18]. Solar radiation data were obtained from the NASA Langley Research Center (LaRC) POWER Project [19].

### 2.3. Yield and Grain Size, Shape and Quality

Grain was harvested using a small plot combine and grain yields were adjusted to 15% moisture content. Harvested grain was cleaned through a 3.5 mm and 2 mm sieve prior to analysis of grain quality. A 25 g sample of grain was measured using a Marvin Seed Analyser (GTA Sensorik GmbH, Wittenburg, Germany) prior to de-hulling for individual grain length (mm), width (mm) and area (mm^2^). The ratio of grain width to grain length was used as an indicator of grain roundness where 0 is very elongated and 1 is perfectly round. Grain hectoliter weight (kg hL^−1^) was measured using a chondrometer (Nileme, C288) on 3 replicate samples (approximately 500 mL) per field plot.

Groat content (often referred to as groat percentage or kernel content) was determined by passing 25 g of whole grain through a Laboratory Oat Huller (Codema Model LH5095; Maple Grove, Minneapolis, MN, USA) set at 100 bar for 60 s and then separating the output into groats and whole grain. Groat content was calculated as:-

Groat Content (%) = 100 × (Groat weight (g)/(Initial weight (g) − Whole grain weight (g)))

Hullability (%) was calculated as:

Hullability (%) = 100 − (100 × Whole grain weight (g)/Initial weight (g))

Thousand grain weight (TGW; g) was determined using the mean weight of 3 samples of 250 seeds. Yield in terms of grain numbers m^−2^ field area was calculated from the grain yield and TGW data.

### 2.4. Grain Composition

Nitrogen (N %) and oil (%) content of groats were predicted using near infrared spectroscopy (NIRS). Protein % was calculated as N × 5.36 [20] Approximately 20 g of cleaned, dehulled groats were scanned in a transport quarter cup cell at 2 nm intervals over the wavelength range 400 to 2498 nm in reflectance mode using a NIRSystems 6500 spectrophotometer (FOSS UK, Warrington, UK). Data were collected using ISI software (Infrasoft International, Port Matilda, PA, USA) and spectra were stored as log 1/R where R is the diffuse reflectance. The calibration equations used for prediction were developed using groat samples originating from multiple harvest years (between 1997 and 2011) and trial sites within the UK and included both spring and winter varieties. Samples to represent the population were selected on the basis of a Mahalanobis-H and neighbourhood-H distances of 3.0 and 0.6, respectively, [21]. Wet chemical analyses for N and oil, were performed on milled (1 mm sieve) subsamples. Nitrogen (%) was determined by a rapid combustion method using a LECO FP-428 analyser (LECO Corp., St. Joseph, MI, USA) and oil (%) was extracted using petroleum ether on a Soxtec system (FOSS UK, Warrington, UK). Calibration equations were developed using WinISI II 1.04a (Infrasoft International LLC, State College, PA) software but standard normal variate (SNV) and de-trending (DT) transformations were applied [22,23] in the order SNV then DT. Cross validation (8 groups) was used to avoid overfitting and to study the robustness of the calibration models and two outlier elimination passes were performed. The final equations were selected on the basis of minimising the standard error of cross validation (SECV) and maximising the coefficient of determination of cross validation (RCV2). Calibration statistics for the equations are shown in Table S1. β-glucan content was determined on a subsample of ground groat using the McCleary method Megazyme™ kit K-BGLU (Megazyme International Ireland Ltd., Wicklow, Ireland) according to AOAC Official Method995.16 [24].

### 2.5. Statistical Analysis

Mean values for grain yield and quality traits for varieties within each environment were analysed by modified joint regression using the RJOINT procedure in Genstat Version 19 [25]. This was used to characterise the sensitivity of varieties to environmental effects by fitting a regression model between the within-environment trait means for the varieties (*V_i_*, *i* = 1…4) and the trait mean within each environment (*E_j_*, *j* = 1,…,22) [26].

*y_ij_* = *V_i_* + *b_i_*
*E_j_* + *error_ij_*

The regression slope (*b_i_*) describes the response of each variety to the environment with higher values reflecting greater sensitivity to the environment.

Pearson’s product-moment correlation coefficients were calculated using within-environment variety means to assess the level of association between the measured traits and using environment means to assess the association between traits meteorological conditions.

A two-dimensional biplot [27,28] based on the first and second principal components was generated to illustrate relative variation within the traits and within the varieties and also associations between traits and between traits and varieties.

## 3. Results

### 3.1. Weather Conditions and Grain Yield

The four growing seasons had very different weather conditions (Table 1) but were representative of oat growing conditions in the United Kingdom. December 2010 to April 2011 was relatively dry (mean of 142 mm cumulative rainfall across the three sites) in contrast to the Western sites in 2014 which received up to 574 mm rain between December and April. Summer 2012 was also very wet across all sites with a mean of 229 mm cumulative rainfall in June and July. Summer 2013 was the driest of the 4 growing seasons with only 63 mm cumulative rainfall in June and July. Mean temperatures in July were also higher in 2013 and 2014 whereas mean temperatures in March were lower in 2011 and 2013 than in 2012 and 2014.

Environment had a significant effect (*p* < 0.001) on both grain number m^−2^ and on grain yield (Table 2) which, averaged over all varieties, ranged from 4.84 to 10.49 t ha^−1^ across the 22 environments. There was no significant difference in yield between varieties or in the sensitivity of varieties to the environment (Table 3).

### 3.2. Grain Physical Quality Characters

There was a significant (*p* < 0.001) effect of environment on thousand grain weight (TGW) which ranged from 35.5 g to 48.2 g across the 22 environments (Table 2) and a significant effect (*p* < 0.001) of variety with the mean TGW of Mascani (45.4 g) significantly greater than Gerald (37.4 g) (Table 3). Varieties differed (*p* = 0.005) in terms of sensitivity of TGW to the environment. TGW was most stable for Mascani (*b_i_* = 0.799) and most sensitive for Balado (*b_i_* = 1.381, Table 3).

Groat content was affected by environment (*p* < 0.001) ranging from 68.65 to 75.88% across the 22 sites (Table 2), by variety (*p* < 0.001, Table 3) and in sensitivity to the environment (*p* < 0.001). Across the 22 environments, Mascani had the highest mean value at 76.78% and was the most stable. Balado was the most sensitive to the environment and had the lowest mean value. Environment affected both hullability (*p* < 0.001) and hectoliter weight (*p* < 0.001). There were also significant differences (*p* < 0.001) between variety means and in their stability (*p* < 0.001) across environments for hullability, with Mascani not only having the highest mean hullability (98.54%) but was the most stable (*b_i_* = 0.129) whilst the other 3 all showed hullabilities differing by greater than 30 percentage units across environments. Interestingly, environment 11, which showed the highest mean hullability and groat content, was the lowest yielding site (Table 2). Significant variety effects (*p* < 0.001) on hectoliter weight were obtained (Table 2 and Table 3) but the varieties did not differ in their sensitivity to environment.

Grain width, grain length and grain roundness all significantly differed (*p* < 0.001) between environments (Table 4) and between varieties with Mascani having the widest grains (Table 5). Varieties also differed (*p* < 0.025) in terms of stability of grain width with Balado the most sensitive to environment and Mascani the most stable (Table 5). Neither grain length nor grain roundness differed in sensitivity to environment between varieties.

### 3.3. Grain Composition

There was a significant effect (*p* < 0.001) of both the variety and environment on protein, oil and β-glucan contents which, averaged over all varieties, ranged from 7.77 to 12.33%, 6.48 to 7.83% and 3.16 to 4.88%, respectively, across environments (Table 4). Balado had the highest mean β-glucan content (4.76%). Neither protein content nor β-glucan content showed any significant difference in sensitivity to the environment between varieties. Sensitivity of oil content however differed between varieties (*p* < 0.004) with Balado being the most sensitive (*b_i_* = 1.450) and Gerald (*b_i_* = 0.786) the least sensitive. Oil content was lowest in Mascani.

### 3.4. Association between Traits

**Figure 1:**
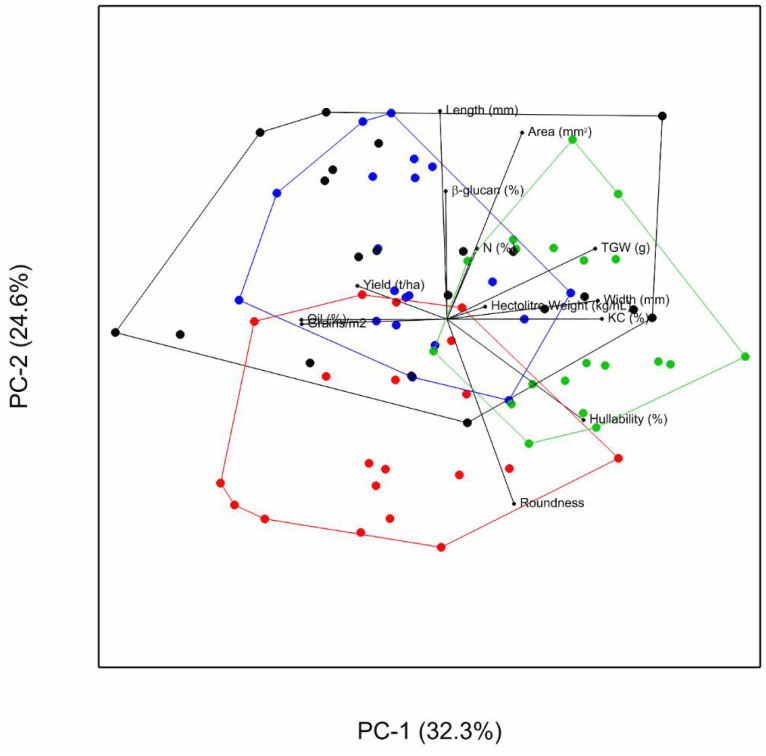
Principal component biplot of mean trait values for 4 winter oat varieties grown in 22 environments. KC: groat content, TGW, thousand grain weight. Balado—Black; Gerald—Red; Mascani—Green; Tardis—Blue.

The relationship between the measured traits is summarized as a biplot in Figure 1. The lengths of the vectors connecting the traits to the biplot origin indicate the relative level of variability in each trait. The angle between the vectors of two traits measures the degree of association between them, acute angles indicating positive correlation and obtuse angle negative correlation. Pearson product-moment correlation coefficients were also calculated between traits (Table S2). Grain yield and grain number m^−2^ were positively correlated (r = 0.91, *p* < 0.001), as were TGW, grain width and groat content. β-glucan content and hullability (r = −0.62, *p* < 0.01), and yield, grain roundness and hullability were inversely correlated The convex hulls illustrate greater between environment variation with Balado in terms of yield, grain number, hectoliter weight, groat content, oil content and grain width than the remaining varieties.

### 3.5. Association between Traits and Environmental Variables

Correlations between site means for each trait and monthly mean temperature, cumulative radiation and cumulative radiation recorded for each environment (excluding the spring sown environment 8) was conducted and the main associations identified are shown in Table 6. A significant negative association between cumulative rainfall from December to April and grain nitrogen content was found (*p* < 0.001). Cumulative rainfall from March until July was negatively associated with yield, grain number m^−2^, hectoliter weight and grain β-glucan content but positively associated with hullability. Grain number m^−2^ and β-glucan content were also positively associated with cumulative radiation in June and the mean temperature in July. Although TGW was not associated with any environmental variable recorded, mean grain width was inversely related to July mean temperature.

## 4. Discussion

Year-to-year variability in temperature, precipitation, and solar radiation is increasing due to global climate change. This enhanced variation will likely lead to more frequent and larger GEI effects impacting milling quality, grain composition and grain yield. Yield gap is a well-known issue in crop production [29]. It refers to the difference between maximal crop yield in ideal trial conditions and actual yield, frequently driven by sub-optimal environmental conditions on farm. Of equivalent importance for many cereals such as oats is the ‘quality gap’ where the threshold levels of grain quality required for acceptance by the milling industry and official reported quality values are often not achieved on-farm.

In this study, carried out over 4 years, there were significant differences between the varieties tested in physical grain quality and composition, across the 22 environments, with environment having a significant effect on all the traits measured. Several approaches can be used to analyze the sensitivity of varieties to the environment [2,6]. In this study, a value of the regression slope from modified joint regression analysis (*b_i_* > 1) indicates varieties with above average sensitivity to environment while those varieties with a slope value (<1) show below average sensitivity, or are more stable. This approach is widely used to quantify adaptation of varieties to environment in plant breeding programs [30] and to analyze the stability of seed yield when multiplied in different environments [31]. It can also help to identify varieties well suited to low input environments. In terms of physical grain characters, hullability and groat content showed a significant GEI indicating that the four varieties differed in their sensitivity with regard to these traits. Both these characters have major implications for mill output and efficiency [7]. Environment had a significant effect on the hullability of all the varieties except for Mascani, which had a hullability of more than 90% in all 22 environments, showing that it was the least sensitive (or most stable) of the four varieties to the environment as regards this trait. Genotype, ontogeny, environment, and agronomic factors have all been considered as factors influencing hullability [7]. The evidence that varieties differ in sensitivity to environment for this trait, with some varieties more stable over environments than others, is important for the milling industry, as careful choice of variety will enable a better prediction of groat yield. In contrast, the hectoliter weight of all varieties responded similarly to environment with a slope value close to 1, indicating that the hectoliter weight of all four varieties was influenced by environment. Although hectoliter weight is considered a poor indicator of groat yield, its ease of measurement means it is routinely used as a measure of grain quality [7]. Mascani had the highest groat content of the four varieties and a specific weight comparable to Gerald. Both groat content and hullability are regarded as a good indicator of mill yield [7] confirming the value of Mascani as a high quality milling variety and its reputation as the best milling oat currently grown in the UK.

Environmental conditions during grain development were important for both grain yield and quality with increased summer rainfall associated with lower yields, lower β-glucan content and lower hectoliter weights. However, hullability was positively associated with both summer rainfall and shorter rounder grain (Table 6). March temperatures, when the crop is in peak tiller production, were also positively associated with grain size parameters and hullability.

Grain size and shape are increasingly being used as non-destructive image analysis tools as indicators of grain milling quality [32,33]. The four varieties within the experiment differed in their size and shape and their sensitivity to the environment. In addition to TGW, four grain shape parameters were measured; area, length, width and roundness. Significant differences were found between the varieties for these parameters. Gerald had the smallest grain, with the narrowest width and shortest length resulting in being the roundest of the four varieties. Tardis and Mascani had the largest grain but contrasted significantly in grain roundness. Mascani grain was wider than Tardis, but Tardis grain was longer than Mascani. Balado appeared to be most sensitive to the environment. Grain width was the characteristic that was most affected by the environment and was significantly lower when July mean temperatures were higher and when radiation in June was lower. Grain number m^−2^ however was positively associated with July temperatures and with June radiation suggesting that more, smaller grains result under such conditions (Table 6). Competition amongst a greater number of grains can result in incomplete grain filling leading to smaller grains [1,34] however no significant relationship was found in the results reported here between TGW and grain number m^−2^. Wide variability exists in individual grain weight in oats, and it is possible that the structure of the grain population, i.e., the relative proportions of primary, secondary and tertiary grain [35,36] also changes as the grain number m^−2^ increases and this is currently being investigated.

Both TGW (and groat content) were positively associated with grain width but less so with grain length. This has also been found in wheat [37] where it has been shown that length is determined early in grain development whereas grain width is determined later at grain filling [38]. The duration of grain filling has been found to be dependent on the temperature following anthesis. In wheat, for example, higher temperatures were found to both shorten the grain filling period and significantly reduce TGW [39].

In terms of grain composition, all three traits studied were influenced by environment and variety. There was also a significant GEI for oil content, but varieties displayed similar responses to the environment for protein and β-glucan content. Simulation studies have shown that environment and cultivar selection are important factors in determining β-glucan levels [40] and confirmed in this study. In this study, the β-glucan content of the grain of all varieties was influenced by the environment, with the level averaged over the four varieties ranging from 2.8 to 4.4%. This is higher than the range reported in some multi-site studies [2] but lower than in others (2.9–6.8%) on a wider range of oat genotypes [5]. Selection of oats with high levels of β-glucan is a major breeding target to provide oats with β-glucan greater than 4% to meet EU and USA health claims [3]. No interaction between genotype and environment was found in this study for β-glucan content which is similar to previous studies [6,9,12,41]. The extent to which the β-glucan content varies in response to the environment will be important for its future exploitation. The relationship between β-glucan and climate is complex, [2,3,5]. In a comparison of oats grown in a Northern maritime climate, β-glucan levels were lower in more northerly locations [42] which may have resulted from higher rainfall and lower temperatures during grain filling and maturation. This was also found in this study with higher temperatures and lower rainfall during grain filling resulting in higher β-glucan content (Table 6).

Although no significant difference was found between the varieties in grain yield, the difference of 5.66 t ha^−1^ between grain yield in the lowest and highest yielding environments was greater than the yield at the lowest yielding environment. This illustrates how yield is dependent on the combination of location and season. Similar results have been found for spring oats [6,9,21]. Grain yield was closely related to grain number m^−2^ as has been found previously for oats and other cereals [43,44,45] with little correlation to grain size or individual grain weight. Grain size however was associated with the grain quality traits measured here (Figure 1, Table S2).

Excess summer rainfall during grain filling resulted in both lower grain number m^−2^ and lower yields (Table 6) partly due to reduction in radiation levels associated with cloudy days. Similar results have been found for maize [46] with excess water causing crop losses of 16% [47]. Excess rainfall may also lead to extended vegetative growth. Although this provides more green leaf area for light capture, cloudy conditions may restrict this. In winter wheat, grain numbers have been shown to be influenced by solar radiation, particularly in the period leading up to anthesis [48].

## 5. Conclusions

Environmental conditions during grain development resulted in significant variation for all traits with significant GEI for groat content, hullability, thousand grain weight, grain width and oil content. Prediction of grain quality for the prospective harvest from environmental conditions encountered during crop development would have considerable benefit to both farmers and to the grain trade. Understanding of varietal response to the environment could also inform breeding decisions.

Breeding programs face a huge challenge in addressing the complexity of factors affecting quality and quantity of yield, and in striking a balance between the two to maximize the value of the crop to the producer. For example, in this study grain yield was negatively related to the hullability of the grain indicating the difficulty of improving both traits simultaneously. Breeders also need to identify genotypes not only with superior performance but that are stable across a range of environments The greatest challenge is to combine grain yield with all milling quality traits in a single variety whilst minimizing GEI of any individual trait [9]. Of the varieties tested here, Mascani was the most stable for all the milling quality traits measured (groat content, hullability, hectoliter weight) and also had the highest mean values for these traits. Maintaining grain quality under a variable climate is critical for human nutrition, end-use functional properties and commodity value.

## Figures and Tables

**Table 1:** Field sites, sowing and harvest dates of the winter oat trials grown across the UK over four harvest years (2011–2014) and environmental conditions during those trials including total rainfall (mm) in the periods specified and mean air temperatures for March and July.

Environment	Site	Latitude (N, °.′)	Longitude (E, °.′)	Sowing Date	Harvest Date	Rainfall Dec to April (mm)	Rainfall June + July (mm)	Mean March Temperature (°C)	Mean July Temperature (°C)
1	Gogerddan	52.43	−4.02	28/09/11	09/08/12	387	273	8.0	13.9
2	Gogerddan	52.43	−4.02	23/10/12	18/08/13	426	91	2.9	16.6
3	Gogerddan	52.43	−4.02	25/09/13	24/07/14	574	96	6.8	15.7
4	Lydbury	52.45	−2.94	30/09/11	22/08/12	270	211	7.9	14.3
5	Lydbury	52.45	−2.94	08/10/13	20/08/14	316	84	6.4	16.2
6	Bidney	52.20	−2.87	15/10/10	17/08/11	145	105	6.1	14.4
7	Rosemaund	52.08	−2.39	06/10/11	05/09/12	325	249	8.1	14.8
8	Rosemaund	51.97	−2.62	06/02/13	03/09/13		56	2.4	17.9
9	Rosemaund	51.98	−2.60	30/09/13	31/07/14	555	88	6.9	16.9
10	ADAS Rosemaund	52.09	−2.39	28/09/10	13/08/11	149	93	6.6	15.0
11	ADAS Rosemaund	52.09	−2.39	27/09/11	06/09/12	325	249	8.1	14.8
12	ORC Elm Farm	52.36	1.35	19/10/10	03/08/11	131	126	6.4	15.3
13	ORC Elm Farm	52.36	1.35	12/10/11	22/08/12	275	168	8.1	16.0
14	ORC Elm Farm	52.36	1.35	16/10/12	24/08/13	292	35	2.7	17.6
15	Glenrothes	56.19	−3.11	28/09/11	24/08/12	205	260	7.0	12.5
16	Glenrothes	56.19	−3.11	02/10/12	14/08/13	317	90	1.4	15.4
17	Glenrothes	56.19	−3.11	26/09/13	04/08/14	360	105	5.2	14.8
18	Devon	50.27	−3.76	03/10/11	28/08/12	420	275	9.0	15.0
19	Devon	50.27	−3.76	20/10/12	13/08/13	442	43	5.3	17.2
20	Devon	50.27	−3.76	07/10/13	31/07/14	565	120	8.3	17.2
21	Essex	51.58	0.41	06/10/11	16/08/12	218	193	8.5	16.0
22	Essex	51.58	0.41	05/10/13	22/07/14	232	94	8.0	17.7

**Table 2:** Effect of environment on the harvested yield (t·ha^−1^), grain quality and thousand grain weight (TGW) of four winter oat varieties grown in 22 environments. Data are presented as the mean of the 4 winter oat varieties in each environment.

Environment	Yield (t·ha^−1^)	Grain Number (1000 m^−2^)	Groat Content (%)	Hullability (%)	Hectoliter Weight (kg·hL^−1^)	TGW (g)
1	6.03	16.03	68.65	90.07	43.14	37.65
2	8.31	19.90	73.45	77.46	50.17	41.89
3	9.31	24.83	68.88	83.44	50.26	37.92
4	8.08	18.20	75.49	93.60	46.01	44.96
5	7.92	16.56	75.48	91.36	53.66	48.18
6	10.02	22.06	75.23	88.32	55.35	45.27
7	6.77	16.78	73.42	98.50	44.47	40.41
8	4.96	12.38	75.73	93.03	50.89	41.40
9	7.18	17.41	74.13	86.82	49.24	41.74
10	10.19	24.16	74.68	71.34	52.94	42.00
11	4.84	10.88	75.88	99.58	51.12	44.55
12	7.98	18.97	73.97	91.34	59.57	42.55
13	7.26	17.85	73.55	93.06	49.65	40.66
14	9.74	23.24	72.91	76.37	51.88	42.04
15	7.61	17.35	73.08	95.55	* na	44.60
16	8.79	22.02	73.22	84.52	52.13	40.23
17	9.69	22.45	72.99	80.28	53.17	43.25
18	6.83	17.86	69.72	97.52	* na	38.40
19	10.49	26.62	72.55	75.76	51.76	39.66
20	9.94	22.94	72.73	83.17	50.99	43.62
21	8.20	20.62	74.36	97.23	42.64	39.47
22	9.43	26.84	70.56	83.90	49.14	35.52
s.e.	0.381	0.950	0.448	1.544	0.787	0.765
Prob	<0.001	<0.001	<0.001	<0.001	<0.001	<0.001

* na, data not available.

**Table 3:** Mean (*V_i_*) harvested grain yield (t ha^−1^) grain quality and thousand grain weight (TGW) of four winter oat varieties grown in 22 environments and sensitivity (*b_i_*) to environment as determined by modified joint regression analysis.

Variety	Yield (t ha^−1^)		Grain Number (1000 m^−2^)		Groat Content (%)		Hullability (%)		Hectoliter Weight (kg hL^−1^)		TGW (g)	
	Mean (*V_i_*)	*b_i_*	Mean (*V_i_*)	*b_i_*	Mean (*V_i_*)	*b_i_*	Mean (*V_i_*)	*b_i_*	Mean (*V_i_*)	*b_i_*	Mean (*V_i_*)	*b_i_*
Balado	7.98	1.151	19.31	1.218	70.82	1.744	83.64	1.522	48.09	1.085	41.81	1.381
Gerald	8.24	0.982	22.16	1.078	72.81	1.096	87.75	1.052	52.16	0.998	37.44	0.988
Mascani	8.18	1.020	18.05	0.912	76.78	0.525	98.54	0.129	52.22	0.926	45.39	0.799
Tardis	8.25	0.844	19.74	0.785	72.23	0.590	81.38	1.290	49.16	0.990	41.90	0.808
s.e.	0.167	0.1046	0.420	0.1022	0.227	0.1108	0.761	0.0957	0.358	0.0907	0.341	0.1184
Prob.	0.625	0.270	<0.001	0.028	<0.001	<0.001	<0.001	<0.001	<0.001	0.691	<0.001	0.005

**Table 4:** Effect of environment on grain dimensions (width, length and roundness) and grain composition (oil,%; protein,% and β-glucan, %). Data are presented as the mean of four winter oat varieties grown in each environment.

Environment	Width (mm)	Length (mm)	Roundness	Oil (%)	Protein (%)	β-Glucan (%)
1	3.10	10.83	0.287	7.82	8.42	3.72
2	3.13	12.79	0.245	7.03	10.40	4.61
3	3.06	10.74	0.286	7.79	7.77	4.70
4	3.30	11.04	0.300	7.33	10.18	3.82
5	3.34	10.69	0.312	7.21	9.54	4.44
6	3.25	11.79	0.276	7.37	9.59	4.35
7	3.19	10.42	0.306	7.21	9.65	3.61
8	3.08	12.58	0.244	6.48	12.11	4.88
9	3.11	11.09	0.281	7.24	8.36	4.60
10	3.16	12.31	0.258	7.66	11.85	4.11
11	3.27	11.10	0.295	6.59	11.10	3.98
12	3.19	10.67	0.300	7.27	10.83	4.19
13	3.18	12.24	0.262	7.00	10.88	3.97
14	3.13	12.18	0.258	7.34	9.70	4.29
15	3.31	10.86	0.306	7.83	9.06	3.35
16	3.08	11.92	0.259	6.99	9.97	4.63
17	3.20	10.79	0.297	7.69	8.42	4.27
18	3.14	10.26	0.307	7.74	8.95	3.16
19	3.08	12.55	0.246	7.59	8.79	4.21
20	3.21	10.98	0.293	7.19	7.88	4.43
21	3.11	10.52	0.297	7.23	10.88	3.74
22	2.94	10.67	0.276	7.70	12.33	4.61
s.e.	0.024	0.113	0.0033	0.104	0.19	0.098
Prob.	<0.001	<0.001	<0.001	<0.001	<0.001	<0.001

**Table 5:** Mean grain dimensions (width, length and roundness) and grain composition (oil, protein and β-glucan). of four winter oat varieties grown in 22 environments and sensitivity (*b_i_*) to environment as determined by modified joint regression analysis.

Variety	Width (mm)		Length (mm)		Roundness		Oil, %		Protein, %		β-Glucan, %	
	Mean (*V_i_*)	*b_i_*	Mean (*V_i_*)	*b_i_*	Mean (*V_i_*)	*b_i_*	Mean (*V_i_*)	*b_i_*	Mean (*V_i_*)	*b_i_*	Mean (*V_i_*)	*b_i_*
Balado	3.16	1.316	11.62	1.030	0.273	0.986	7.53	1.450	9.92	1.056	4.76	1.101
Gerald	3.09	0.952	10.53	0.866	0.294	0.890	7.36	0.786	9.54	0.956	3.57	0.986
Mascani	3.23	0.817	11.23	1.066	0.290	1.142	6.70	0.906	9.76	1.011	4.26	1.068
Tardis	3.17	0.901	11.89	1.038	0.268	0.982	7.72	0.817	10.13	0.977	4.07	0.843
s.e.	0.011	0.113	0.050	0.063	0.0014	0.0666	0.047	0.127	0.09	0.065	0.043	0.097
Prob.	<0.001	0.025	<0.001	0.119	<0.001	0.074	<0.001	0.004	<0.001	0.729	<0.001	0.263

**Table 6:** Pearson correlation coefficients between rainfall (mm) and site means for grain yield, quality and composition (excluding spring sown trial, environment 8).

Variable	December to April Cumulative Rainfall		March to July Cumulative Rainfall		June Cumulative Rainfall		June and July Cumulative Rainfall		November Mean Monthly Temperature		March mean Monthly Temperature		July Mean Monthly Temperature		June Cumulative Radiation	
Yield (t ha^−1^)	−0.042		−0.772	***	−0.788	***	−0.768	***	−0.448	*	−0.422		0.404		0.467	*
Groat content (%)	−0.441	*	−0.179		−0.217		−0.137		−0.385		−0.121		−0.062		−0.361	
Hullability (%)	−0.169		0.758	***	0.739	***	0.803	***	0.454	*	0.612	**	−0.481	*	−0.485	*
Hectoliter weight ^$^ (kg hL^−1^)	−0.234		−0.715	***	−0.545	*	−0.611	**	−0.625	**	−0.414		0.110		0.170	
Grain number m^−2^	0.039		−0.680	***	−0.718	***	−0.698	***	−0.296		−0.339		0.492	*	0.551	**
TGW (g)	−0.222		−0.099		−0.072		−0.065		−0.357		−0.116		−0.266		−0.312	
Grain width (mm)	−0.268		0.249		0.278		0.313		−0.050		0.164		−0.531	*	−0.516	**
Grain length (mm)	−0.059		−0.477	*	−0.560	**	−0.539	*	−0.202		−0.634	**	0.297		0.103	
Grain roundness	−0.047		0.526	*	0.612	**	0.607	**	0.175		0.634	**	0.472	*	0.287	
Grain protein (%)	−0.727	***	−0.174		−0.122		−0.002		−0.171		0.015		0.080		−0.254	
Grain β-glucan (%)	0.260		−0.730	***	−0.800	***	−0.846	***	−0.633	**	−0.553	**	0.605	**	0.594	**
Grain oil (%)	0.109		−0.114		0.006		−0.031		−0.004		0.187		−0.139		0.290	

*** *p* < 0.001, ** *p* < 0.01, * *p* < 0.05; 19 degrees of freedom. ^$^, 17 degrees of freedom.

## Data Availability

The data presented in this study are available on request from the corresponding author.

## Supplementary Materials

The following are available online at https://www.mdpi.com/article/10.3390/foods10102356/s1, Table S1: NIR Calibration statistics: Table S2: Pearson Correlation coefficients between site means for grain yield, quality, dimensions and composition.
