# Supplementary material for: Genotype and Environment Affect the Grain Quality and Yield of Winter Oats (Avena sativa L.)
Source: Foods. 2021 Oct 3;10(10):2356. doi: 10.3390/foods10102356 (PMC8535078; doi:10.3390/foods10102356)
Supplement: Supplementary file 1 [file foods-10-02356-s001.zip › foods-1369155-supplementary.pdf]

Table S1 NIR Calibration statistics

Equation	n	Mean	Minimum	Maximum	F	SEC	R^2	SECV	R_{CV}^2
Oil (%)	1132	7.35	2.60	16.93	11	0.43	0.94	0.46	0.93
Nitrogen (%)	1108	1.87	1.03	3.21	11	0.09	0.94	0.09	0.94

n = number of samples in calibration; F = number of factors in calibration model; SEC = standard error of calibration;

R^2 = squared correlation coefficient; SECV = standard error of cross calibration; R_{CV}^2 = squared correlation coefficient (cross validation)

Table S2 Pearson Correlation coefficients between site means for grain yield, milling quality, grain dimensions and composition *** $p<0.001$, ** $p<0.01$

	**Grain yield**	**Groat Content**	**Hullability**	**Hectoliter weight**	**Grain number m^{-2}**	**TGW**	**Grain width**	**Grain length**	**Grain roundness**	**Grain Nitrogen**	**Grain β-Glucan**	**Grain oil**
Grain yield	1.00											
Groat Content	-0.02	1.00										
Hullability	-0.77***	0.12	1.00									
Hectoliter weight	0.39	0.29	-0.37	1.00								
Grain number m^{-2}	0.93***	-0.30	-0.75***	0.21	1.00							
TGW	-0.02	0.78***	0.12	0.44	-0.39	1.00						
Grain width	-0.27	0.64**	0.41	0.23	-0.59**	0.91***	1.00					
Grain length	0.40	0.25	-0.65***	0.27	0.33	0.08	-0.12	1.00				
Grain roundness	-0.47	0.02	0.74***	-0.16	-0.52	0.28	0.49	-0.92***	1.00			
Grain Nitrogen	-0.07	0.39	0.05	0.06	-0.01	-0.07	-0.09	0.22	-0.23	1.00		
Grain B-Glucan	0.50	0.08	-0.62**	0.53	0.48	0.04	-0.33	0.38	-0.49	-0.01	1.00	
Grain oil	0.37	-0.66***	-0.28	-0.05	0.48	-0.37	-0.30	-0.31	0.16	-0.36	-0.12	1.00
